# Impact of the triglyceride-glucose index on prognosis following endovascular therapy for acute ischemic stroke: effect modification by collateral circulation

**DOI:** 10.3389/fneur.2026.1838009

**Published:** 2026-06-12

**Authors:** Yishu Wang, Qinyu Lei, Jiaqi Wang, Yachao Lin, Liguo Xu

**Affiliations:** Department of Interventional Radiology, The Second Affiliated Hospital of Zhengzhou University, Zhengzhou, China

**Keywords:** acute ischemic stroke, collateral circulation, endovascular therapy, functional outcome, TyG index

## Abstract

**Objective:**

To investigate the association between the triglyceride-glucose (TyG) index and 90-day functional outcome after endovascular therapy in patients with acute ischemic stroke, to evaluate the risk assessment performance of the TyG index combined with clinical variables, and to assess the effect-modifying role of collateral circulation in this association.

**Methods:**

A retrospective analysis was conducted in 209 patients with acute ischemic stroke who underwent endovascular therapy at our hospital between October 2022 and December 2025. Multivariable logistic regression was performed to evaluate factors associated with poor functional outcome, defined as a modified Rankin Scale (mRS) score > 2 at 90 days. Interaction and stratified analyses were conducted to assess whether collateral circulation modified the association between the TyG index and clinical outcomes. Receiver operating characteristic (ROC) curves and the corresponding area under the curve (AUC) were used to evaluate the predictive performance of the TyG index combined with clinical variables. Differences in AUCs were compared using DeLong’s test. Calibration was assessed using the Hosmer-Lemeshow test, Brier score, and calibration curve, and internal validation was performed with 1,000 bootstrap resamples.

**Results:**

A total of 209 patients were included, with 145 (69.4%) in the good functional outcome group (modified Rankin Scale [mRS] score≤2) and 64 (30.6%) in the poor functional outcome group (mRS > 2). Univariate analysis showed that the TyG index was significantly higher in the poor functional outcome group, and that sex, baseline National Institutes of Health Stroke Scale (NIHSS) score and collateral circulation status also differed significantly between the two groups (all *p* < 0.05). Multivariable logistic regression analysis showed that the TyG index was independently associated with poor functional outcome at 90 days after endovascular therapy (OR = 1.972, *p* < 0.001). A significant interaction was observed between the TyG index and collateral circulation (*p* = 0.012), suggesting a possible effect-modifying role of collateral circulation, and stratified analysis showed that this association appeared to be more pronounced in patients with poor collateral circulation. The combined risk assessment model showed good discriminative ability for poor functional outcome at 90 days (AUC = 0.834, sensitivity = 0.828, specificity = 0.731, DeLong’s test: *p* < 0.001), and the addition of the TyG index further improved the discriminative ability and assessment performance of the model.

**Conclusion:**

The TyG index is an independent risk factor for poor 90-day functional outcome after endovascular therapy in patients with acute ischemic stroke. Collateral circulation may modify this association. The TyG index also improves the risk assessment performance of the combined risk model.

## 1 Introduction

Acute ischemic stroke is characterized by hypoxic–ischemic injury to brain tissue and remains associated with high morbidity and mortality (1). Current national and international guidelines recommend endovascular therapy as early as possible for eligible patients with acute ischemic stroke (2). However, previous studies have shown that a substantial proportion of patients fail to achieve functional independence at 90 days even after recanalization therapy, including 46–55% of those treated within the therapeutic time window and 41–43% of those treated beyond it (3). Therefore, accurate early risk stratification and identification of high-risk patients are critical for clinical decision-making and management.

Insulin resistance (IR) is an important metabolic abnormality and has been widely recognized as an early predictor of cardiovascular events and an independent risk factor for adverse vascular outcomes (4). However, direct measurement of IR is complex and not readily applicable in routine clinical practice. The triglyceride-glucose (TyG) index has recently emerged as a simple and reliable surrogate marker of insulin resistance, with high sensitivity and specificity for identifying vascular and metabolic abnormalities (5). It is calculated as ln [fasting triglycerides (mg/dL) × fasting blood glucose (mg/dL)/2] (6). In patients with acute ischemic stroke, an elevated TyG index has been associated with an increased risk of stroke and with unfavorable outcomes, including stroke recurrence, all-cause mortality, and early neurological deterioration (7, 8). However, the association between the TyG index and functional outcome after endovascular therapy in patients with acute ischemic stroke has not been fully elucidated.

In the pathophysiology of acute ischemic stroke, collateral circulation serves as an important hemodynamic compensatory mechanism (9), directly influencing the survival of the ischemic penumbra and final infarct volume (10). In addition, collateral circulation is a key determinant of functional outcome after endovascular therapy (11). Digital subtraction angiography (DSA) is considered the gold standard for collateral assessment, and collateral status can be quantitatively evaluated using the ASITN/SIR grading system (12). Previous studies have shown that patients with good collateral circulation (ASITN/SIR grade 3–4) have higher rates of successful recanalization and better functional outcome (13).

Taken together, functional outcome after acute ischemic stroke is likely influenced by both systemic metabolic status and vascular hemodynamic factors. The association between the TyG index and outcome after endovascular therapy may differ across patient subgroups, and collateral circulation may have a possible effect-modifying role in this relationship. Therefore, this study aimed to investigate the association between the TyG index and functional outcome after endovascular therapy in patients with acute ischemic stroke, and to further assess the potential modifying role of collateral circulation status on this association. Integrating metabolic and hemodynamic factors may provide additional information for clinical risk assessment.

## 2 Materials and methods

### 2.1 Study population

Inclusion criteria were as follows: (1) acute ischemic stroke diagnosed on the basis of clinical manifestations and imaging findings; (2) eligibility for and completion of endovascular therapy according to the European Stroke Organization–European Society for Minimally Invasive Neurological Therapy guidelines (14); (3) successful recanalization after endovascular therapy, defined as a modified Thrombolysis in Cerebral Infarction (mTICI) grade ≥2b; (4) availability of preoperative DSA images of adequate quality; (5) availability of preoperative fasting venous blood samples for measurement of triglycerides (TG) and fasting plasma glucose (FPG); and (6) complete clinical and imaging data, including a 90-day modified Rankin Scale (mRS) score (15). Exclusion criteria were as follows: (1) concomitant cerebral hemorrhage; (2) severe comorbid conditions with a life expectancy of less than 3 months; (3) a history of major neurological disease or a baseline mRS score ≥2; and (4) incomplete clinical or imaging data.

This was a single-center retrospective study. A total of 217 patients were initially screened, and 8 were subsequently excluded because of missing data. Ultimately, 209 eligible patients with acute ischemic stroke admitted to the Second Affiliated Hospital of Zhengzhou University between October 2022 and December 2025 were included.

### 2.2 Research methods

#### 2.2.1 Baseline characteristics

Baseline characteristics included demographic, clinical, imaging, and laboratory variables. Demographic variables included age and sex. Clinical variables included systolic blood pressure, diastolic blood pressure, body mass index (BMI), stroke etiology at admission according to the TOAST criteria, National Institutes of Health Stroke Scale (NIHSS) score at admission, medical history (including hypertension, diabetes mellitus, atrial fibrillation, prior stroke, and smoking), and onset-to-reperfusion time, defined as the interval from symptom onset to reperfusion. Imaging variables included preoperative DSA findings, including the occluded vessel territory (internal carotid artery, middle cerebral artery, or vertebrobasilar artery) and collateral circulation status assessed using the ASITN/SIR grading system, with grades 0–2 defined as poor collateral circulation and grades 3–4 as good collateral circulation. Laboratory variables included triglycerides (TG), fasting plasma glucose (FPG), total cholesterol (TC), low-density lipoprotein cholesterol (LDL-C), and high-density lipoprotein cholesterol (HDL-C). The TyG index was calculated as ln [fasting triglycerides (mg/dL) × fasting plasma glucose (mg/dL)/2].

#### 2.2.2 Collateral circulation assessment

Collateral circulation status was assessed using the American Society of Interventional and Therapeutic Neuroradiology/Society of Interventional Radiology (ASITN/SIR) collateral grading system. Preoperative DSA images were independently reviewed by two neuroimaging experts who were blinded to clinical outcomes. According to the ASITN/SIR grading system, grades 0–2 were classified as poor collateral circulation and grades 3–4 as good collateral circulation (16).

#### 2.2.3 Outcome assessment

The primary outcome was 90-day functional outcome after endovascular therapy. Follow-up was conducted at 90 days after treatment by telephone interview or medical record review. According to the mRS, scores of 0–2 were classified as a good functional outcome, whereas scores of 3–6 were classified as a poor functional outcome (17).

### 2.3 Statistical analysis

Continuous variables are presented as mean ± standard deviation (SD) when normally distributed and as median with interquartile range (IQR) when non-normally distributed. Categorical variables are presented as frequencies and percentages [*n* (%)]. According to the modified Rankin Scale (mRS) score at 90 days, patients were classified into a good functional outcome group (mRS ≤ 2) and a poor functional outcome group (mRS > 2). Differences between the two groups were compared using the chi-square test for categorical variables, the independent-samples *t*-test for normally distributed continuous variables, and the Mann–Whitney *U* test for non-normally distributed variables. Univariate logistic regression analysis was performed to evaluate the associations between candidate variables and poor functional outcome, and odds ratios (ORs) with 95% confidence intervals (CIs) were calculated.

Based on univariate analyses, variables were selected for inclusion in the multivariable logistic regression model to estimate odds ratios (ORs) and their 95% confidence intervals (95% CIs), aiming to identify independent risk factors associated with poor functional outcomes. Collinearity among the included variables was assessed using variance inflation factors (VIFs). Additionally, to evaluate the robustness of the model, a sensitivity analysis was conducted using penalized logistic regression poor functional outcome Receiver operating characteristic (ROC) curves and the corresponding area under the curve (AUC) were used to evaluate the discriminatory performance of relevant factors and the combined model for functional outcome after endovascular therapy. The optimal cutoff value was determined using the Youden index, and the corresponding sensitivity and specificity were calculated. Differences in AUCs between models were compared using DeLong’s test. The calibration performance of the risk assessment model was further evaluated using the Hosmer–Lemeshow goodness-of-fit test, Brier score, and calibration curve. Internal validation was performed using 1,000 bootstrap resamples to assess the stability of model performance. To assess effect modification, an interaction term between the mean-centered TyG index and collateral circulation status was introduced into the adjusted multivariable logistic regression model. Stratified analyses were further performed according to collateral circulation status, and multivariable logistic regression analyses were conducted separately in patients with good and poor collateral circulation.

All statistical analyses were performed using SPSS version 27.0 and R version 4.4.1. All tests were two-tailed, and *p* < 0.05 was considered statistically significant.

## 3 Results

### 3.1 Baseline characteristics and univariate analysis

A total of 209 patients with acute ischemic stroke were included, of whom 145 were classified into the good functional outcome group (mRS ≤ 2) and 64 into the poor functional outcome group (mRS > 2). Compared with the good functional outcome group, the poor functional outcome group had significantly higher admission NIHSS scores and TyG index values (both *p* < 0.001), as well as a higher proportion of poor collateral circulation (75.0% vs. 21.4%, *p* < 0.001). In addition, the proportion of male patients, prevalence of hypertension, and levels of total cholesterol and low-density lipoprotein cholesterol were significantly higher in the poor functional outcome group (*p* = 0.005, *p* = 0.002, *p* = 0.003, and *p* < 0.001, respectively). Stroke etiology according to the TOAST classification also differed significantly between the two groups (*p* = 0.030). No significant between-group differences were found in age, weight, height, BMI, high-density lipoprotein cholesterol, systolic blood pressure, diastolic blood pressure, diabetes mellitus, onset-to-reperfusion time, smoking history, history of atrial fibrillation, history of stroke, or occluded vessel territory (all *p* > 0.05). Detailed baseline characteristics are shown in Table 1.

**Table 1:** Baseline characteristics of the 209 patients according to functional outcome.

Variable	Good functional outcome (mRS ≤ 2) *n* = 145	Poor functional outcome (mRS > 2) *n* = 64	t/Z/*χ*^2^	*p-*value
Age (years)	64.17 ± 10.93	63.89 ± 10.23	0.175	0.861
Weight (kg)	67.92 ± 8.69	70.06 ± 9.34	−1.603	0.111
Height (m)	1.67 (1.61, 1.72)	1.68 (1.62, 1.72)	−0.444	0.657
BMI (kg/m^2^)	24.48 ± 3.13	25.11 ± 3.39	−1.316	0.190
Systolic blood pressure (mmHg)	147.00 (132.00, 163.00)	140.50 (129.25, 157.50)	−1.683	0.092
Diastolic blood pressure (mmHg)	87.57 ± 11.40	84.47 ± 12.90	1.738	0.084
Admission NIHSS score (points)	14.00 (12.00, 17.00)	16.50 (13.50, 19.00)	−3.375	<0.001
Total cholesterol (mmol/L)	3.49 (2.88, 4.04)	4.03 (3.28, 4.50)	−3.005	0.003
Low-density lipoprotein cholesterol (mmol/L)	2.20 ± 0.75	2.58 ± 0.78	−3.374	<0.001
High-density lipoprotein cholesterol (mmol/L)	1.03 (0.86, 1.25)	1.04 (0.87, 1.28)	−0.150	0.881
TyG index	8.26 (7.74, 9.08)	9.67 (8.80, 10.94)	−6.474	<0.001
Onset-to-reperfusion time (h)	8.50 ± 1.34	8.87 ± 1.73	−1.522	0.129
Sex			7.715	0.005
Female	50 (34.5%)	10 (15.6%)		
Male	95 (65.5%)	54 (84.4%)		
Hypertension			9.142	0.002
No	71 (49.0%)	17 (26.6%)		
Yes	74 (51.0%)	47 (73.4%)		
Diabetes mellitus			2.139	0.144
No	99 (68.3%)	37 (57.8%)		
Yes	46 (31.7%)	27 (42.2%)		
History of atrial fibrillation				1.000
No	139 (95.9%)	61 (95.3%)		
Yes	6 (4.1%)	3 (4.7%)		
History of stroke			0.655	0.418
No	96 (66.2%)	46 (71.9%)		
Yes	49 (33.8%)	18 (28.1%)		
Smoking history			3.643	0.056
No	95 (65.5%)	33 (51.6%)		
Yes	50 (34.5%)	31 (48.4%)		
Stroke etiology (TOAST classification)				0.030
Large-artery atherosclerosis	115 (79.3%)	43 (67.2%)		
Cardioembolism	11 (7.6%)	14 (21.9%)		
Other determined etiology	6 (4.1%)	1 (1.6%)		
Undetermined etiology	13 (9.0%)	6 (9.4%)		
Occluded vessel territory			1.883	0.390
Internal carotid artery	78 (53.8%)	32 (50.0%)		
Middle cerebral artery	32 (22.1%)	11 (17.2%)		
Vertebrobasilar artery	35 (24.1%)	21 (32.8%)		
Collateral circulation			54.299	<0.001
Good	114 (78.6%)	16 (25%)		
Poor	31 (21.4%)	48 (75%)		

Data are presented as mean ± standard deviation (SD) for normally distributed continuous variables, median with interquartile range (IQR) for non-normally distributed continuous variables, and frequencies and percentages [*n* (%)] for categorical variables. Between-group comparisons were performed using the independent-samples t test, Mann–Whitney *U* test, or chi-square test, as appropriate. BMI, body mass index; NIHSS, National Institutes of Health Stroke Scale.

Univariate logistic regression analysis showed that admission NIHSS score, total cholesterol, low-density lipoprotein cholesterol, TyG index, male sex, hypertension, and poor collateral circulation were significantly associated with poor functional outcome (all *p* < 0.05). Detailed results are shown in Table 2.

**Table 2:** Univariate analysis of functional outcome in 209 patients.

Variable	OR	95% CI	*p*-value
Age	0.998	0.970–1.025	0.860
Weight	1.028	0.994–1.063	0.112
Height	2.643	0.042–165.995	0.645
BMI	1.064	0.970–1.167	0.190
Systolic blood pressure	0.990	0.997–1.003	0.126
Diastolic blood pressure	0.978	0.953–1.003	0.085
Admission NIHSS score	1.144	1.051–1.245	0.002
Total cholesterol	1.609	1.136–2.279	0.007
Low-density lipoprotein cholesterol	1.944	1.296–2.916	0.001
High-density lipoprotein cholesterol	0.923	0.325–2.623	0.881
TyG index	2.324	1.769–3.054	<0.001
Onset-to-reperfusion time	1.168	0.956–1.426	0.129
Sex (male)	2.842	1.334–6.057	0.007
Hypertension (1)	2.653	1.394–5.047	0.003
Diabetes mellitus (1)	1.571	0.856–2.882	0.145
History of atrial fibrillation (1)	1.139	0.276–4.705	0.857
History of stroke (1)	0.767	0.402–1.460	0.419
Smoking history (1)	1.785	0.982–3.246	0.058
Stroke etiology (TOAST classification)			0.035
Occluded vessel territory			0.393
Collateral circulation (1)	11.032	5.527–22.021	<0.001

Dichotomous variables were coded as binary variables: sex (male = 1, female = 0), hypertension (yes = 1, no = 0), diabetes mellitus (yes = 1, no = 0), history of atrial fibrillation (yes = 1, no = 0), history of stroke (yes = 1, no = 0), smoking history (yes = 1, no = 0), and collateral circulation status (poor = 1, good = 0). BMI, body mass index; NIHSS, National Institutes of Health Stroke Scale.

### 3.2 Multivariable logistic regression analysis of poor functional outcome

To avoid multicollinearity and model overfitting, and based on clinical relevance and evidence from the literature, only six variables—sex, hypertension, baseline NIHSS score, onset-to-reperfusion time, TyG index, and collateral circulation status—were included in the multivariable logistic regression analysis. Male sex (OR = 10.459, *p* < 0.001), hypertension (OR = 3.195, *p* = 0.011), admission NIHSS score (OR = 1.190, *p* = 0.004), and TyG index (OR = 1.972, *p* < 0.001), and poor collateral circulation (OR = 17.788, *p* < 0.001) were identified as independent risk factors. Detailed results are shown in Table 3. Variance inflation factors (VIFs) were calculated for all included independent variables, with a maximum value of 1.240, indicating no significant multicollinearity. The results of the penalized regression sensitivity analysis were consistent with those of the primary analysis.

**Table 3:** Multivariable logistic regression analysis of poor functional outcome in 209 patients.

Variable	OR	95% CI	*p*-value
Sex (1)	10.459	3.321–32.938	<0.001
Hypertension (1)	3.195	1.303–7.835	0.011
Admission NIHSS score	1.190	1.058–1.337	0.004
Onset-to-reperfusion time	1.075	0.802–1.441	0.628
TyG index	1.972	1.381–2.815	<0.001

Binary variables were coded as follows: sex (male = 1, female = 0), hypertension (yes = 1, no = 0), and collateral circulation status (poor = 1, good = 0). NIHSS, National Institutes of Health Stroke Scale.

### 3.3 Interaction analysis between the TyG index and collateral circulation

Before constructing the interaction term, the TyG index was mean-centered to reduce potential multicollinearity between the main effects and the interaction term. The interaction term between the mean-centered TyG index and collateral circulation was then included in the multivariable logistic regression model, with adjustment for sex, hypertension, admission NIHSS score, onset-to-reperfusion time, the mean-centered TyG index, and collateral circulation status. Collinearity diagnostics showed that the maximum VIF value was 2.358, indicating no serious multicollinearity. The interaction term between the mean-centered TyG index and collateral circulation was statistically significant (*β* = 1.058, OR = 2.880, 95% CI: 1.283–6.466, *p* = 0.010), suggesting that collateral circulation may modify the association between the TyG index and poor functional outcome. Detailed results are shown in Table 4.

**Table 4:** Multivariable logistic regression analysis of the interaction between the TyG index and collateral circulation.

Variable	*β*	OR	95% CI	*p*-value
Sex (1)	2.479	11.934	3.263–43.648	<0.001
Hypertension (1)	1.319	3.741	1.430–9.787	0.007
Admission NIHSS score	0.166	1.181	1.046–1.334	0.007
Onset-to-reperfusion time	0.119	1.127	0.826–1.537	0.452
Mean-centered TyG index	0.204	1.227	0.731–2.060	0.364
Collateral circulation (1)	2.759	15.787	5.674–43.928	<0.001
Mean-centered TyG index × collateral circulation	1.058	2.880	1.283–6.466	0.010

Binary variables were coded as follows: sex (male = 1, female = 0), hypertension (yes = 1, no = 0), and collateral circulation status (poor = 1, good = 0). NIHSS, National Institutes of Health Stroke Scale.

### 3.4 Stratified analysis by collateral circulation status

In the stratified analysis, 130 patients were included in the good collateral circulation group, among whom 16 experienced poor functional outcomes; 79 patients were included in the poor collateral circulation group, among whom 48 experienced poor functional outcomes. Stratified analysis suggested that the association between the TyG index and poor functional outcome appeared to differ according to collateral status. The association was weaker and did not reach statistical significance in patients with good collateral circulation (*p* = 0.383), whereas a stronger association was observed in patients with poor collateral circulation (*p* < 0.001). These findings further suggest that collateral circulation may modify the association between the TyG index and outcome. Detailed results are shown in Table 5.

**Table 5:** Stratified multivariable logistic regression analysis by collateral circulation status.

Variable	Good collateral circulation group *n* = 130,events = 16 OR/95% CI	*p*-value	Poor collateral circulation group n = 79,events = 48 OR/95% CI	*p*-value
Sex (1)	4.799/0.590–39.032	0.142	20.602/3.781–112.255	<0.001
Hypertension (1)	1.77/0.558–5.613	0.332	11.802/2.158–64.542	0.004
Admission NIHSS score	1.175/0.998–1.383	0.053	1.203/0.987–1.467	0.067
TyG index	1.246/0.760–2.043	0.383	4.622/2.060–10.369	<0.001

Binary variables were coded as follows: sex (male = 1, female = 0) and hypertension (yes = 1, no = 0). NIHSS, National Institutes of Health Stroke Scale. Events, poor functional outcome, defined as a 90-day modified Rankin Scale (mRS) score >2.

### 3.5 Development and performance evaluation of the combined risk model

**Figure 1:**
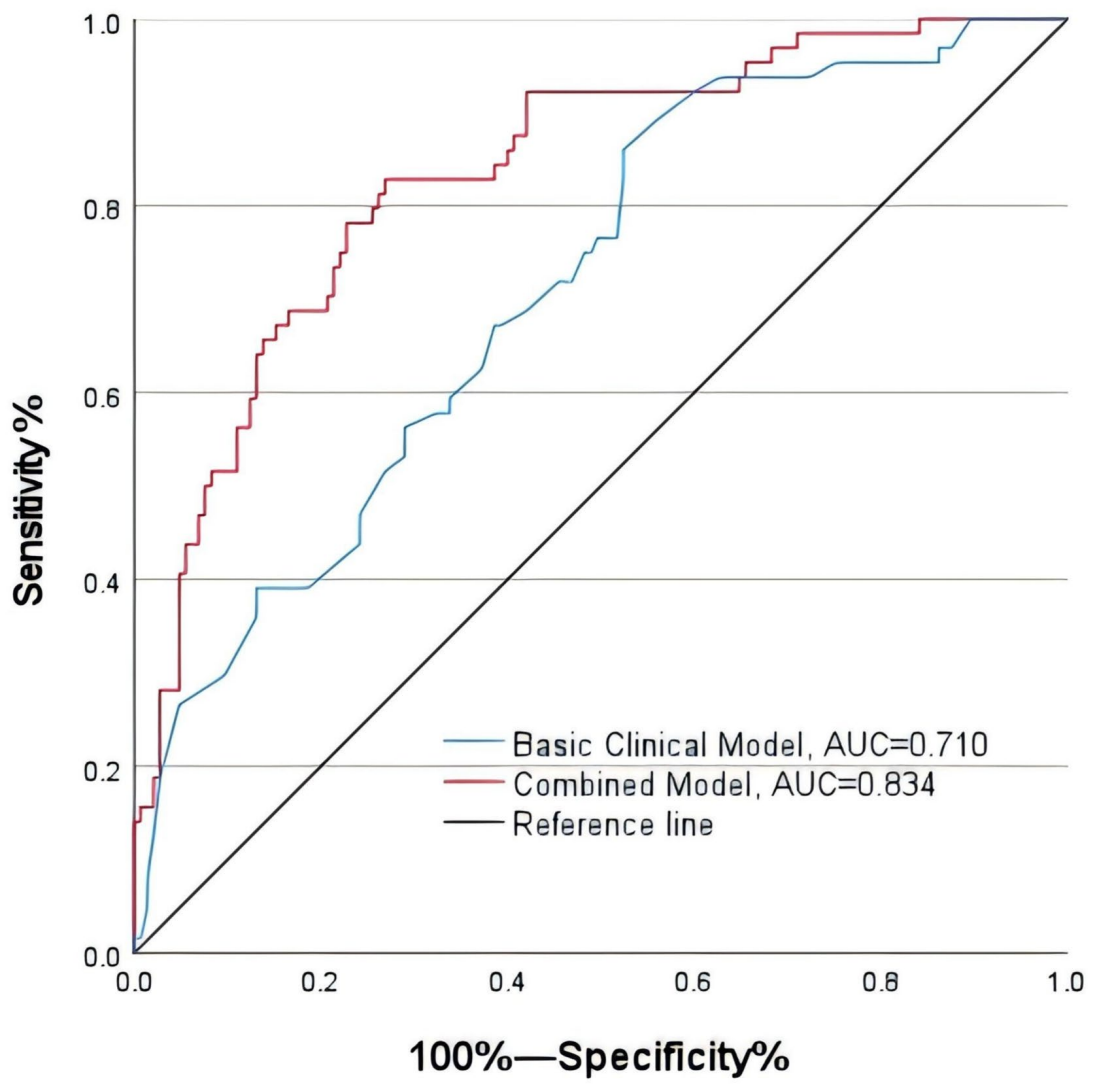
ROC curves of the basic clinical model and combined model for assessing the risk of poor functional outcome in 209 patients.

To evaluate the incremental value of the TyG index in assessing the risk of poor functional outcome, two risk models were constructed. The basic clinical model included sex, hypertension, and admission NIHSS score, whereas the combined model was developed by additionally incorporating the TyG index into the basic clinical model. ROC curve analysis showed that the basic clinical model yielded an AUC of 0.710, with a sensitivity of 0.859 and a specificity of 0.476, whereas the combined model yielded an AUC of 0.834, with a sensitivity of 0.828 and a specificity of 0.731. DeLong’s test showed that the AUC of the combined model was significantly higher than that of the basic clinical model, with a statistically significant difference (AUC difference = 0.134, 95% CI: 0.054–0.196; *Z* = 3.437; *p* < 0.001). This finding suggests that the TyG index further improved the discriminative ability of the model beyond the basic clinical variables. The multivariable logistic regression equation for assessing the risk of poor functional outcome at 90 days was as follows: logit(P) = −12.589 + 0.912 × TyG index + 0.119 × admission NIHSS score + 0.990 × hypertension + 1.345 × male sex. Hypertension was coded as yes = 1 and no = 0, and male sex as male = 1 and female = 0. The predicted probability was calculated as $p=\frac{1}{1+e^{-\text{Logit}(P)}}$ . The model can be used to assess the risk of poor functional outcome at 90 days after endovascular treatment in patients with acute ischemic stroke. Detailed results are presented in Figure 1 and Table 6. Considering that collateral circulation is an important imaging-based hemodynamic factor influencing stroke outcome and was also investigated as an effect modifier in this study, collateral circulation was further added to the combined model to construct an extended model. The results showed that the AUC of the extended model increased to 0.893 after incorporating collateral circulation. DeLong’s test demonstrated that the AUC of the extended model was significantly higher than that of the combined model (*p* = 0.009), suggesting that the addition of collateral circulation further improved the discriminative ability of the model.

**Table 6:** ROC analysis results of the basic clinical model and the combined model for assessing the risk of poor functional outcome in 209 patients.

Variable	AUC	95% CI	Optimal cutoff value	Sensitivity	Specificity
Basic clinical model (predicted probability)	0.710	0.636–0.783	0.217	0.859	0.476
Combined model (predicted probability)	0.834	0.776–0.893	0.280	0.828	0.731

**Figure 2:**
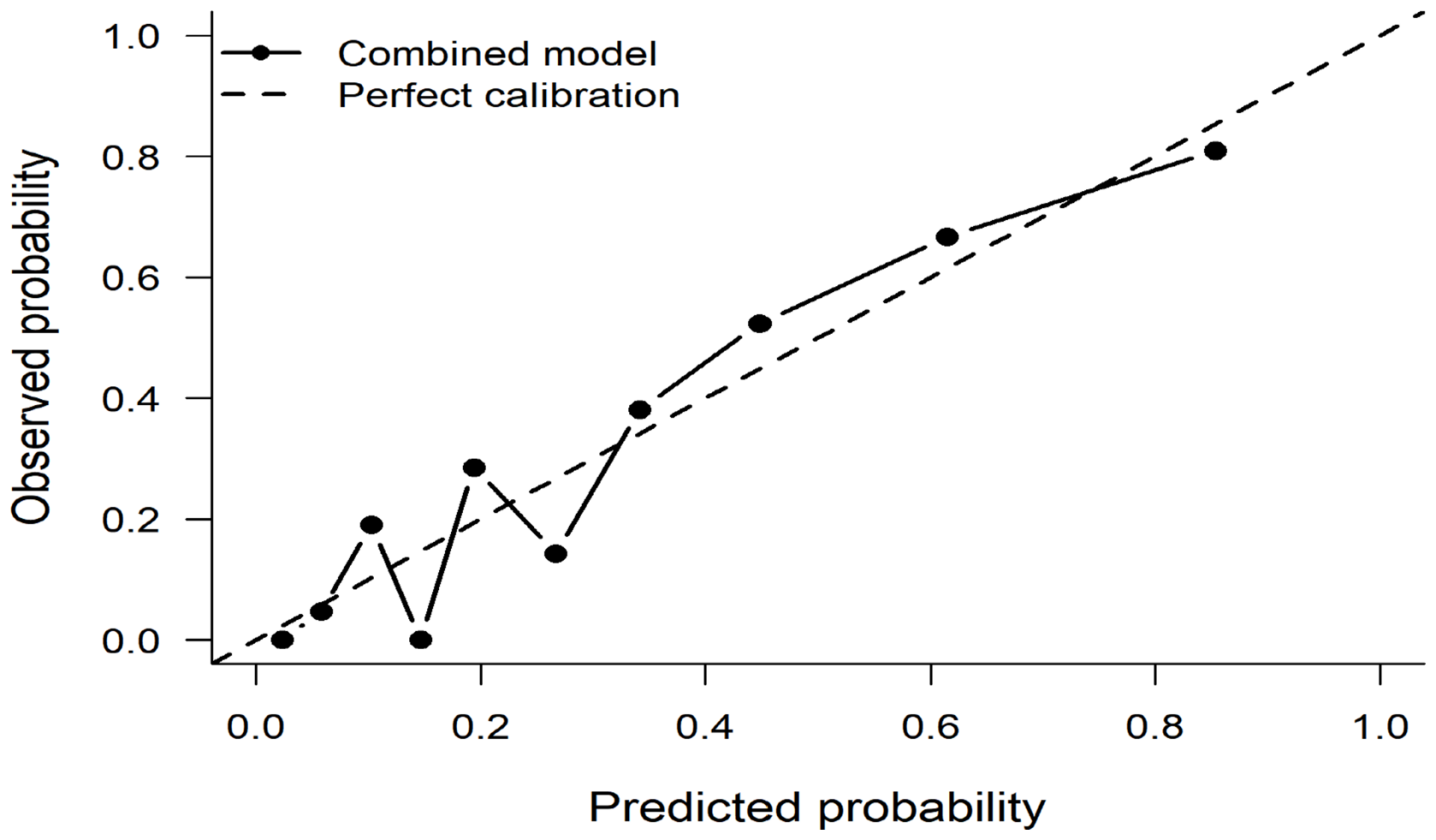
Calibration curve of the combined risk assessment model.

Further calibration assessment and internal validation were performed for the combined risk assessment model. The Hosmer-Lemeshow goodness-of-fit test showed no significant lack of fit for the model (*χ*^2^ = 9.860, df = 8, *p* = 0.275). The Brier score was 0.148, indicating a relatively low overall assessment error. The calibration curve demonstrated overall agreement between the model-estimated probabilities and the observed probabilities. Internal validation using 1,000 bootstrap resamples showed a bootstrap mean AUC of 0.842, with a 95% CI of 0.775–0.896, suggesting stable discriminative ability of the combined model. The calibration curve is shown in Figure 2.

## 4 Discussion

In this study, clinical data from 209 patients with acute ischemic stroke who underwent endovascular therapy were analyzed. We systematically evaluated the association between the TyG index and 90-day functional outcome after endovascular therapy and further explored the effect-modifying role of collateral circulation. The results showed that the TyG index was independently associated with poor functional outcome at 90 days. Further interaction and stratified analyses suggested that collateral circulation may modify the association between the TyG index and outcome, with a more pronounced adverse association observed in patients with poor collateral circulation. In addition, a combined risk assessment model was constructed using the TyG index together with common clinical variables, and this model showed improved performance in assessing the risk of poor functional outcome. Insulin resistance is an important pathophysiological basis of metabolic disorders and is closely associated with obesity, diabetes mellitus, hypertension, dyslipidemia, inflammation, and atherosclerosis (18). It contributes to atherosclerotic progression, endothelial dysfunction, inflammatory activation, and thrombosis (19) and has also been linked to an increased risk of ischemic stroke (20). Therefore, effective assessment of insulin resistance is of considerable importance for early cardiovascular risk identification (4). The TyG index, a composite indicator derived from fasting triglyceride and glucose levels (21), has recently been proposed as a simple and readily available surrogate marker of insulin resistance (22). Although the mechanisms underlying the association between the TyG index and outcome in ischemic stroke remain unclear, this relationship may be partly explained by insulin resistance and disordered glucose and lipid metabolism. Elevated triglyceride levels usually reflect disordered lipid metabolism, which is closely related to insulin resistance (23). In addition, hyperglycemia can impair the biological effects of insulin through multiple metabolic pathways, thereby reducing insulin sensitivity (24). Hyperglycemia has also been associated with a higher likelihood of poor functional outcome in acute ischemic stroke compared with normoglycemia (25). In patients undergoing endovascular therapy, these metabolic abnormalities may be associated with ineffective recanalization or unfavorable outcome despite successful recanalization.

Previous evidence from patients with chronic total coronary occlusion has shown that hyperinsulinemia is associated with impaired coronary collateral circulation, suggesting that insulin resistance-related metabolic abnormalities may adversely affect collateral development (26). Although this study focused on coronary rather than cerebral collaterals, it provides indirect but relevant support for the biological plausibility of our findings. This study further suggests that collateral circulation may play an effect-modifying role in the association between the TyG index and outcome. In acute ischemic stroke, collateral circulation is considered an important compensatory mechanism that influences preservation of the ischemic penumbra, final infarct volume, and ultimately functional outcome (27). Good collateral circulation can provide additional perfusion to ischemic tissue, delay ischemic injury, and extend the therapeutic window for endovascular therapy, whereas poor collateral circulation reflects insufficient perfusion compensation and renders brain tissue more vulnerable to infarction (28). In patients with a high TyG index, pathological processes such as insulin resistance, atherosclerosis, endothelial dysfunction, and inflammatory activation may partly explain the observed adverse association between the TyG index and outcome, especially under conditions of hypoperfusion and reperfusion injury. This effect may be more pronounced in patients with poor collateral circulation (20, 27, 29). The interaction and stratified analyses further supported the possible effect-modifying role of collateral circulation at the statistical level, suggesting that collateral circulation was not merely a confounding factor but more likely acted as an effect modifier in the association between the TyG index and outcome. These findings indicate that the association between the TyG index and outcome after endovascular therapy may differs according to collateral circulation status. Patients with poor collateral circulation showed a stronger adverse association between elevated TyG index and poor functional outcome, which may provide additional information for refined clinical risk stratification.

Previous studies have shown that a higher TyG index is associated with an increased risk of poor functional outcome in patients with acute ischemic stroke (29). A retrospective study further reported that the TyG index was an independent but moderate predictor of poor functional outcome at 3 months after stroke (30). Another study found that the TyG index in patients with ischemic stroke was significantly associated with poor functional outcome at discharge and increased in-hospital mortality (31). A recent cross-sectional study in acute ischemic stroke also reported an association between the TyG index and collateral status, indicating that insulin resistance, as reflected by the TyG index, may serve as a predictor of poorer collateral circulation (32). Based on these findings, the present study investigated the association between the TyG index and 90-day outcome after endovascular therapy in patients with acute ischemic stroke and further constructed a combined risk assessment model. Compared with baseline clinical variables alone, incorporation of the TyG index improved the risk assessment performance of the model. In addition, collateral circulation, as an indicator of cerebrovascular hemodynamics, was further included in interaction and stratified analyses. The results suggested that collateral circulation may modify the association between the TyG index and outcome. These findings provide a new perspective for integrating metabolic status with hemodynamic characteristics in clinical risk assessment. It should be noted that the combined model was used as the primary risk assessment model in this study, while an extended model incorporating collateral circulation was further constructed as a supplementary analysis. This approach does not deny the clinical significance of collateral circulation, but rather reflects the analytical purpose and model positioning of the present study. Specifically, the primary model was designed to evaluate the incremental assessment value of the TyG index beyond routine clinical variables, whereas collateral circulation was mainly investigated as an effect modifier of the association between the TyG index and outcome, which has been specifically evaluated through interaction and stratified analyses. Therefore, collateral circulation was not included in the primary risk assessment model; instead, an extended model was constructed to further assess its supplementary contribution to model performance.

This study has several limitations. First, as a single-center retrospective study, it may have been subject to selection bias. Several important imaging variables related to endovascular treatment, including ASPECTS score, infarct core volume, and occlusion site, were not included because of incomplete imaging data and the retrospective study design, and residual confounding related to these factors cannot be completely excluded. Second, the sample size was limited, particularly in the interaction and stratified analyses, where some subgroups were relatively small, resulting in wide confidence intervals and potentially reduced statistical power. Accordingly, these findings require further validation in larger studies. In addition, the combined risk model developed in this study has not undergone external validation, and its stability and generalizability remain to be confirmed. Finally, the TyG index was calculated from fasting blood glucose and triglyceride levels at admission, which may have been influenced by the stress response and may therefore have limitations as a surrogate marker of insulin resistance. Nevertheless, our findings may provide a basis for future multicenter prospective studies.

## 5 Conclusion

In summary, the TyG index was independently associated with 90-day functional outcome in patients with acute ischemic stroke after endovascular therapy, and collateral circulation may modify this association. In addition, a combined risk assessment model constructed using the TyG index together with common clinical variables showed improved discriminative ability and assessment performance for evaluating the risk of poor functional outcome at 90 days. However, given the retrospective single-center design and the relatively limited sample size, these findings should be interpreted with caution, and further validation in prospective multicenter studies is warranted.

## Data Availability

The raw data supporting the conclusions of this article will be made available by the authors, without undue reservation.
